# Engaging future healthcare professionals for rural health services in South Africa: students, graduates and managers perceptions

**DOI:** 10.1186/s12913-021-06178-w

**Published:** 2021-03-12

**Authors:** Dumsani M. Gumede, Myra Taylor, Jane D. Kvalsvig

**Affiliations:** School of Nursing and Public Health Medicine, College of Health Sciences, University of KwaZulu-Natal, Durban, South Africa

**Keywords:** Healthcare professionals, Non-profit organization, Improving healthcare systems, Rural health, Recruitment and retention system, KwaZulu-Natal, South Africa

## Abstract

**Background:**

The shortage of healthcare professionals (HCP) negatively affects health services in rural areas in many parts of the world, as is the case in South Africa. Innovative programs designed to improve the recruitment strategies for health system in a rural area are essential. They need support with a scholarship and mentorship programme for young people from rural areas to study for health science degrees, with the aim that they would take up a post at the hospital in their community, once qualified.

This paper reports the perceptions and experiences of the students and graduates sponsored by the foundation, and those of managers from the facilities where the students were ultimately placed, in order to gauge whether such a programme can make a sustainable contribution to address the shortage of health personnel in rural areas and to what extent this is happening.

**Methods:**

The authors used qualitative methods, combining semi-structured in-depth interviews and focus groups and the data were analyzed thematically.

**Results:**

The results provide information on students interviewed who appreciated the financial and socio-emotional support that they received. On the other hand, graduates value the availability of jobs in their home community on completion of their studies. The managers reported the success of the programme in increasing the number of healthcare personnel at the hospitals, and the increased range of available medical services. Since the graduates are familiar with the language and culture of their patients the managers considered that they are better able to assist them.

**Conclusions:**

The system was well thought-out and achieved its goal of improving health services in an underdeveloped rural area of South Africa. More could be achieved if other government services in the area were simultaneously improved and if the system were replicated elsewhere. The students and graduates from rural areas are involved on sustaining health services in rural areas while rural managers support the programme and make suggestions for improvement and to promote the program in other regions.

**Supplementary Information:**

The online version contains supplementary material available at 10.1186/s12913-021-06178-w.

## Background

The United Nations has proposed the Sustainable Development Goals in order to improve universal health coverage for all people and ensure quality of health coverage at all places including rural areas [1], although this is difficult due to the inability of rural areas to attract and retain healthcare professionals (HCP) due to a number of factors [2]. Therefore, the search for innovative strategies that involve communities is very important for ensuring health.

Many South Africans living in rural areas lack access to affordable, quality, and comprehensive health care, despite significant government investment in programmes to strengthen the health system. As in many other countries in the sub-Saharan region, rural areas have a high burden of disease, high levels of unemployment, and poor healthcare services at public health facilities [3–6], and the problem is worsened by the shortage of health workers [7]. In 2013 South Africa had 60 doctors per 100,000 population, compared to a global average of 152/100,000 [8].

Fifteen per cent of poor rural households in South Africa live more than an hour away from the closest clinic and 20% live more than an hour away from the closest hospital [9, 10]. Transport is expensive and unreliable, and roads are in poor condition, so that the costs of accessing health services can be prohibitive [11]. It is not easy to attract health personnel to rural areas for reasons which include the lack of good schools and social amenities. Non-profit organisations (NPO) assist the Department of Health by rendering technical assistance, training at facility level, and other services but few address the shortage of trained staff in rural health facilities [12].

This paper presents the strategy of an organization, the Umthombo Youth Development Foundation (UYDF) whose goal is to attract and retain health workers in rural health facilities. This programme was based on evidence from Australia and Canada showing that students of rural origin are more likely to work in rural areas than those from urban areas [13, 14]. The programme commenced in January 1999 in an area which lacked good health and education services, and where unemployment was high [15].

The UYDF addressed the shortage of healthcare workers in the provinces of KwaZulu-Natal and the Eastern Cape, by identifying young people who were eligible for scholarships in health sciences. The programme commenced with four students and by the end of 2017 had produced 336 graduates and was supporting 251 students with an annual pass rate of over 90%.

The intervention involves an integrated model of recruitment at school level, selection by a local hospital, comprehensive financial support, a compulsory structured academic and social mentoring programme, and experiential holiday work at the hospitals [16]. Upon completion of their degrees, graduates are absorbed into the hospitals where they were initially interviewed for the scholarship. The selected students sign a year-for-year work-back contract with UYDF. The potential cost-savings to the health system are considerable and are detailed elsewhere [17, 18]. This paper is part of series of papers and this paper presents the qualitative section of the records regarding the changes to health service delivery and in the lives of the participants and the community. The quantitative aspects will be presented in another paper. The objective of this paper is to explore the views of students, graduates and hospital managers at rural hospitals hosting graduates and students, on the contribution of the UYDF programme to rural public hospitals.

## Methods

The study design was descriptive and used qualitative methods. A phenomenological approach was used to understand the experiences of the students and graduates who had been supported by the UYDF. The aim of using this approach was to describe the meaning of both students’ and graduates’ experiences of the support provided by the UYDF during their training, in terms of their experiences and how they perceived the UYDF support [19, 20]. Using qualitative methods, interviews and focus group discussions were used to collect the data from each of the three groups, namely, the current students (interviews), the graduates (focus group discussions) and the hospital managers (interviews) and interview and focus group discussion guides were developed for this study (see Table 1, below). We used a critical action research approach, having worked as a mentor to the students supported by the UYDF. This approach deepened the understanding of the mentoring process and the perceptions of both students and the mentor. In order to guard against bias which might arise from the participants being interviewed by a former UYDF mentor, experienced Social Science Research Assistants (SSRA) from the Africa Health Research Institute interviewed the students and the hospital management participants, transcribed their responses, translating them from IsiZulu to English, when necessary. Focus group discussions were also held with graduates to explore the similarities and differences in their experiences.

**Table 1:** Description of Study Participants

Category	Interviewees (numbers per category)	Description	Type of data collection
Students currently supported by UYDF (*n* = 50)	Bachelor of Medicine (MBChB) (23), pharmacy (7), physiotherapy (5), radiography (4), and then 2 each for audiology, nursing, occupational therapy, optometry and speech therapy, and dental therapy (1)	There were 29 (58%) females & 21 (42) males who participated in the study, Medical students were between there 2nd and 5th year of study. The other students were 2nd or 3rd year students.	Individual interviews (IDIs) were held.
Graduates at rural hospitals who had qualified as a result of UYDF support (*n* = 25)	The Graduates included the professions of medicine (3), pharmacy (3), physiotherapy (3), nursing (1), dietetics (2), occupational therapy (3), dentistry (2), radiography (2), optometry (1), audiology (2), speech therapy (1), psychology (1) and social work (1).	Of the graduates *n* = 19 were women, of whom 11 were unmarried.	Three focus group discussions (FGDs) were held
Managers at rural hospitals where HCPs were based, and students worked in their holidays (*n* = 14)	The management included Chief Executive Officers [CEO] (2), Medical Managers (6), Human Resource Managers (1), Human Resource Practitioners (2) and Assistant Directors (District Human Resource) (3) who work closely with the UYDF in the placement of graduates and the identification of hospitals within that district where there are shortages.	Two of the three districts in KwaZulu-Natal where UYDF works were visited and managers from 7 of the 13 hospitals where UYDF operates participated.	Individual interviews (IDIs) were held

### Ethical considerations

Prior to data collection, permission to interview the UYDF scholarship students was given by the UYDF and to interview Department of Health personnel was received from the KwaZulu-Natal Department of Health. The study was given ethical clearance by the University of KwaZulu-Natal’s Humanities and Social Science Ethics Committee. The gatekeeper’s letter, given by the Umthombo Youth Development Foundation (UYDF) director, gave permission to use the records of the UYDF for publication. Participants were assured that their participation in the study would not affect their participation in the UYDF scheme, and that they could withdraw from the study at any time. The participants were informed that the data would not be used for any other purpose than that described in the study information sheet, that all identifying information would be deleted or disguised when the findings of the study were reported to ensure anonymity and confidentiality. Audio-recordings would be stored safely during the study and destroyed when the research was complete. Prior to the interviews, all participants gave a written consent to participate in the study and participation was voluntary as no participant was coerced. Where the author had been a mentor to students, ensuring anonymity was particularly relevant so that students would feel free to state their views without prejudice, because of their reliance on the organisation for financial and other support.

This paper forms part of a larger study examining the contribution of the UYDF programme towards strengthening health systems in rural areas.

### Mentorship

The UYDF management realised that UYDF students lacked emotional and social support which often leads to failure to progress and even to students dropping out altogether. The mentorship programme for all the students was introduced to help the students develop strategies to deal with the heavy workload associated with health science training [21]. UYDF students are allocated mentors that they meet on a monthly basis. The mentoring begins from the first month at university up to the time when the students graduate. This is supplemented by biannual visits by a UYDF senior mentor, orientation, monthly telephone/SMS/email enquiries, quarterly reports by each student, holiday work (practical exposure to patients in June/July and December/January), quarterly peer support at university and the annual Imbizo – year-end meetings to discuss crucial issues with all UYDF funded students [21]. The UYDF believes that providing compulsory mentorship will bridge the gap of poor schooling and equip them with the necessary skills to cope with the demands of Institutions of Higher Learning and health science training [16].

### The participants: students, graduates, and management

#### Students

The study used purposive sampling in order that the sample would “yield the most relevant and plentiful data” [22]. A shortlist of 50 students was created on the basis that the sample would include representatives of all the disciplines supported by the UYDF scholarship scheme and years of study. The purpose was to obtain a broad range of perspectives [23]. They were all full-time students at a South African University and receiving full financial support from UYDF.

The students were from ten health science disciplines with 23 enrolled for a Bachelor of Medicine (MBChB), pharmacy (7), physiotherapy (5), radiography (4), and then 2 each for audiology, nursing, occupational therapy, optometry and speech therapy, and one dental therapist. The MBChB students were between the second and fifth years of academic study, and the others between the second and third academic year.

#### Graduates

Three focus group discussions were held at hospitals which have participated in the programme for many years. The graduates from the UYDF scholarship scheme were all living in the areas where they grew up and completed their schooling, and they had returned after graduation to serve their rural communities at the local rural hospital. The 25 FGD participants were pre-dominantly women (19), 11 were single and the others married with children or engaged to be married. They represented a variety of disciplines: medicine (3), pharmacy (3), physiotherapy (3), nursing (1), dietetics (2), occupational therapy (3), dentistry (2), radiography (2), optometry (1), audiology (2), speech therapy (1), psychology (1) and social work (1).

#### Management

Participants came from two of the three district offices concerned with the intervention, and seven of the 16 hospitals. In order to maximize the diversity of views participants included different health officials with some experience of the UYDF operations, Chief Executive Officers, Medical Managers, Human Resource Managers, and District Human Resource Development Officers who work closely with the UYDF in the placement of graduates and the identification of hospitals within that district where there are shortages, The Human Resource Managers also provide continuous development for the graduates both as a retention strategy and for the general improvements of their skills.

### Data collection

#### Individual interviews

In-depth interviews were used in preference to focus group discussions, so that the experiences relating to social, emotional or financial issues would be captured and thus convenience sampling was used. Using an interview guide, students were asked what they knew about the UYDF, and what benefits they had received/were currently receiving. They were also asked how the UYDF affected their personal lives and that of their families. The interview explored their lives prior to receiving the UYDF support and what they would otherwise have done at the end of their schooling. The interview also explored their future plans and the likelihood of their fulfilling their commitment to serve their communities. As health science students have many commitments during the academic year, the interviews took place at the annual “*Imbizo*” (meeting to discuss issues) at the end of the year. Students participating in the research were called from the meeting and interviewed individually. Interviewees did not have the opportunity to discuss the questions amongst themselves because they were all interviewed during the same session of the meeting. To further ensure confidentiality the interviewers came from a different area and had no previous knowledge of the students.

#### Focus group discussions (FGDs) with the graduates working at rural hospitals

Convenience sampling was used as FGDs were conducted during working hours. During the discussions, the graduates were asked to comment on their lives before they started their studies and the experiences that characterised their years as undergraduates. They were asked about changes that took place in their lives after they had graduated, and what contribution they had made to the institution where they worked. Finally, they were asked to comment on whether they thought the UYDF intervention addressed the shortage of health care professionals in rural areas, and what recommendations regarding this they would make to the Department of Health. Probes were used to clarify the responses of the participants. The FGDs were conducted in isiZulu, at each selected hospital with two SSRAs facilitating and taking notes. Data were transcribed and translated by the SSRAs.

#### Management interviews

Participating officials were interviewed individually and privately at their place of work by the SSRAs during the course of one morning, so there was no opportunity to discuss the topic with colleagues. A purposive sampling approach was used. The interviews were semi-structured to allow participants to speak freely. In total fourteen respondents were interviewed concerning their views on the impact that the UYDF programme had on previous staff shortages and on the quality of hospital services in the area.

### Data analysis

A qualitative thematic analysis model [22], was used to investigate the responses of the students. This enabled the textual data to be synthesised into a meaningful account identifying commonalities and overlapping themes in the subjective constructions [24], using a critical realist framework [23, 25]. This limited bias in the literature and the researcher’s pre-existing beliefs regarding the value of the UYDF scheme.

A preliminary reading of all transcripts was followed by line by line coding to generate the initial codes and emerging themes. Similar themes were then grouped and arranged according to a hierarchy. A ‘theme map’ was created through an editing process which clarified the meaning of the themes and their relationship to one another. Finally, the theme map and the categorisation of the themes was discussed with an independent coder and consensus was reached over the relationship between themes. Patterns were identified through a rigorous process of data familiarisation, data coding, and theme development. The trustworthiness of the data was assessed following Guba’s model of trustworthiness (1981) which outlines credibility, transferability, dependability, and confirmability as the criteria for assessing qualitative research [26–29].

## Results

As well as the intended improvement in the staff complement at rural hospitals, the transcripts documented how graduates benefitted personally, and how their families and communities benefitted. The authors tried to balance respondents’ gender where possible but there were more women than men (58%) and all were black Africans. The average age 22.02 years, range 18–28 years for students. See Table 2 below:

**Table 2:** The participants views (students, graduates and management)

**Students views**	
**Main Themes**	**Quotes from IDI’s**
**Students**	
1. The poor quality of schooling in rural areas	‘… I had never seen a computer, let alone used one and when I came here, I was told to type in assignments …., so it is difficult – you end up handing in assignments late’ ‘I have never done experiments in my life …. But my lecturer expected me to be able to do the experiment and produce the report …. (*Pharmacy Student, 22 years, Female, Hospital B*)
	“In my first year, I hadn’t done chemistry in eleven years [of schooling] … … I was struggling in this module and when I went to consult my chemistry lecturer, I was told if I didn’t understand the basic concepts of chemistry then I chose the wrong career … I am doing my second year I repeated my second year …. They gave me another chance although I had to explain why I failed …” (*Pharmacy Student, 24 years, Female, Hospital C*)
2. Rural-urban migration	“… there are not even traffic lights in KwaNongoma and when you come here there are a thousand of them …., you just wonder how to cross this road.” (*Medical Student, 21 years, Female, Hospital B*)
	“During all my basic education I used one classroom, or if we had to move, we were escorted by a teacher … … when I got to university, I was now expected to move from one lecture hall to another ….” (*Physiotherapy Student, 20 years, Female, Hospital A*)
	“In my first week at university I missed almost all the morning classes as I was expecting to hear the bell ring, but this was not the case here …” (*Audiology Student, 20 years, Male, Hospital B*)
3. The UYDF intervention	“… given such an opportunity all I can do is study and pass and become the best radiographer …. This is not just bursary, the programme offers more, and we are like a family.” (*Female Student, 23 years, Female, Hospital A*)
4. Home Away from Home	“… Umthombo [UYDF] is like home to me. Any time you can call if you have a problem with money, Dumsani [the chief mentor] is there to take you through and they always help you ….” (*Medical Student, 30 years, Male, Hospital C*)
5. Post study placement anxiety	“…. I would say the UYDF made a good plan by signing the MOU [memorandum of understanding] with Department of Health, because as UYDF graduates we have no problem of finding jobs, unlike many graduates in South Africa” (*Medical Student, 28 years, Male, Hospital A*) “…. People who graduate are not guaranteed jobs in South Africa. UYDF graduates are kind of lucky because of the MOU with the Department of Health that tries to place the UYDF graduates back in their rural hospitals without any interviews ….” (*Medical Student, 26 years, Male, Hospital B*)
**Graduates Views**	
**Main Themes**	**Quotes from FGD’s**
**Graduates**	
1. The impact of the UYDF intervention on the personal lives	‘… I come from a poor family and I never thought my family could afford tertiary education for me …. Without UYDF, I would not have made it this far ….’ (*Graduate, 42 years, Male, Hospital A*)
	‘… as a student I would budget my money and sometimes buy a few things for my siblings but now that I am working life has changed and whatever my family needs, I make sure I provide it. That’s how good UYDF has been for me...’ (*Graduate, 27 years, Female, Hospital A)* “…. I love my community that much; I want to see it prosper and the only person to bring that change is me …’ (*Graduate, 31 years, Female, Hospital C*)
	‘… We are willing to start a fund where we can put as little as R100 in future it will help other students who are in the same situation as we were …. Even if it goes towards their studies in other professions … …’ (*Graduate, 39 years, Male, Hospital B*)
2. The Impact of UYDF at Community level	‘… there are some things that my rural home people cannot explain clearly to a white doctor or nurse because they do not know how to say it in their language … .it is now easy to talk to us because they are able to say it in isiZulu’ (*Graduate, 28 years, Female, Hospital B*)
	‘… it is no longer necessary for people in the rural areas to have appointments scheduled in the peri-urban / urban parts of the region because now you also find an optometrist and audiologist at a rural hospital which was usually not the case before …’, (*Graduate, 31 years, Female, Hospital A*)
	‘… Umthombo (UYDF) has afforded me an opportunity which my family was not able to because I come from a very poor background …, now children in my community will see the importance of education.’ ‘… I have built my mother a house and I now drive …., most people in my community also wish to do the same for their families as well as for themselves’. (*Graduate, 35 years, Male, Hospital A*)
	‘… Umthombo (UYDF) should go out there into schools and make the staff and students know the services they provide, what programmes they sponsor, all that information is not readily available …’ (*Graduate, 29 years, Female, Hospital A*)
3. Difficult Workplace Environment	‘… as much we want to stay long in our rural hospitals, but we sometimes feel that we are not appreciated if you look at the type of accommodation that is available within the hospital’ … … it is difficult to work in a place where you are not appreciated’. (*Graduate, 25 years, Female, Hospital B*)
	‘…. the rural community also deserves better and quality health care, as this is stipulated by the constitution of South Africa’ …. sometimes there are essential posts that are not available at district hospital ….’ ‘In my first year at work I had to work the whole year without proper equipment … this affected the provision of quality services as I had to compromise a lot’ (*Graduate, 30 years, Female, Hospital B*)
	‘The accessibility of health care facilities in rural areas still remains (a problem), there is not much of choice in relation to health care facilities and if they are there, they are isolated ….’ (*Graduate, 28 years, Female, Hospital C*) ‘Transport – the availability of transport is a major challenge in our area, the roads are terrible and sometimes not usable ….’ (*Graduate, 26 years, Female, Hospital B*)
**Management views**	
**Main Themes & Sub-Themes**	**Quotes from IDI’s**
**Staffing**	
1. Awareness of UYDF Services	“We were told by UYDF staff that there is a MoU between the department and them (UYDF), but no one has ever explained these agreements to us as the hospitals, and we always get graduates allocated to our hospital by the province ….”. (*HRM, DC A, 41 years, Female*) “If the province can give details on the graduates they are allocating to us, if we knew they were from UYDF, that will help us allocate their duties, and let the UYDF Graduates do more of the outreach programmes because of their local knowledge of the area and community”. (*Assistant Director, DC B, 3 years, Female*)
2. Addressing shortage changes due to the UYDF programme	“When I started here there was only one doctor and one clinical associate with 6 wards to be covered (Male, Female, Peadiatrics, Maternity, OPD and Casualty). Patients used to go back home untreated, and sometimes the ward rounds would only be done once a week in order to cover all wards and that increased the death rate at our hospital,” (*Acting Medical Manager, DC C, 32 years, Male*) “… Our facilities have struggled in the past to train and attract professionals especially health care professionals, due to the lack of funding and the geographical location …” (*HRM/Acting CEO, DC C, 51 years, Male*)
	“… Majority of the healthcare professionals at our institution are there through UYDF. They are long-term staff that bring stability”. (*CEO, DC C, 50 years, Male*)
3. Retention	“… UYDF assists with our staff retention strategy as the students work in their rural communities after completion of their studies, and the majority of them stay longer than their contracts ….”. (*Medical Manager, DC B, 39 years, Female*)
	“…. Even those who came to our facilities, we are unable to retain them, they are overworked with no recreational facilities around our hospitals …” (*Acting Medical Manager, DC C, 32 years, Male*)
**Services**	
1. Better range of services and cutting costs	“…it is no longer necessary for people in the rural areas to have appointments scheduled in the regional or tertiary hospitals…. because patients get a variety of services at the local hospital, such as psychological services, eye care and all rehabilitation services are on site now, which was not the case before…”, (*Medical Manager, DC B, 51 years, Male*)
2. Improved communication, saving lives	“They are generally local and know the language and situation of the patients”. (*Medical Manager, DC B, 39 years, Female*) “Majority of the UYDF graduates had a strong sense of commitment to the rural communities where they work, and they showed responsibility in giving back to their communities of origin ….” (*Medical Manager, DC B, 51 years, Male*) “No one wants to see anyone die, and an idea that fights mortality is always a good idea which needs to be supported. This is exactly what the UYDF is doing for our institution – it prevents deaths of many people”. (*Deputy Director, DC C, 36 years, Male*)

### Health science students

**Fig. 1:**
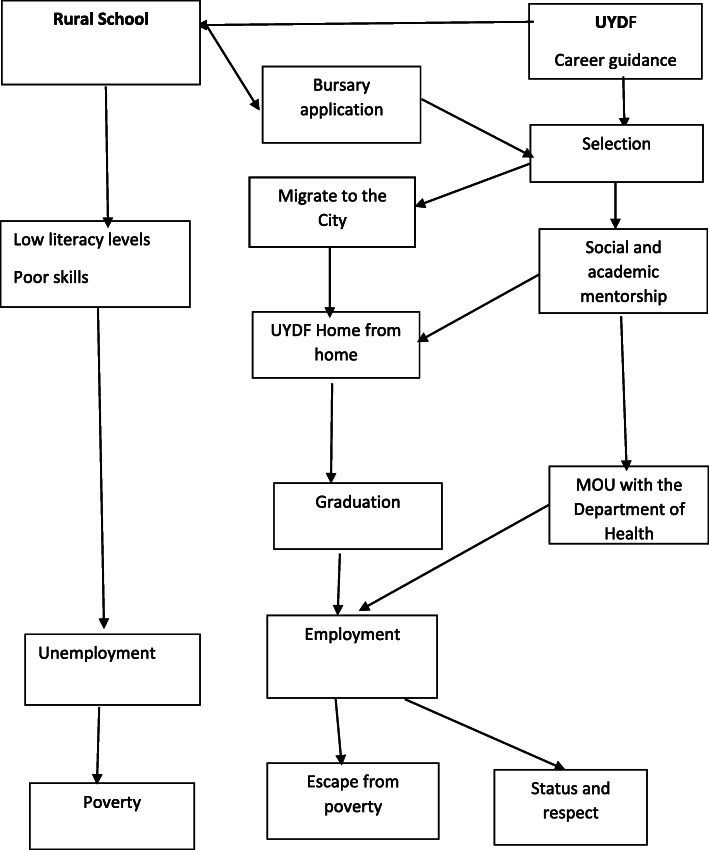
Students views on their experiences as beneficiaries of the UYDF intervention and their hopes for the future

There were more women than men (58%), and all were young black Africans (average age 22.02 years, range 18–28 years). Their responses centred around two main themes, namely: (1) the poor quality of their education at school which would normally lead to poor prospects of being employed (this refers to prior to their selection by UYDF), (2) the UYDF intervention which would lead to a career that they could be proud of. Figure 1 illustrates the way in which the themes and subthemes were connected in a sequential pattern showing the life-changing consequences that the UYDF intervention would have for them. The themes and sub-themes emerged during the analysis (see Fig. 1 below).

### The poor quality of schooling in rural areas

Many rural schools lack facilities, such as computers, libraries and laboratories for those learners who want to pursue science subjects. The sub-themes of ‘Low Levels of Literacy’, ‘Poor Skills’ and ‘Unlikely to find employment’ were identified in the analysis as the key consequences of poor schooling. Many of the students said they were ill-prepared for higher education as a consequence of poor facilities in rural schools. In most South African universities, the medium of instruction is English, and as first language isiZulu speakers this was said to affect their ability to express themselves and shook their confidence.

*‘ … I had never seen a computer, let alone used one and when I came here, I was told to type in assignments …*. , *so it is difficult - you end up handing in assignments late’*

*‘I have never done experiments in my life …*. *but my lecturer expected me to be able to do the experiment and produce the report ….* (Student).

Several students pointed out that rural schools usually do not give guidance concerning the correct subject choice for their careers in the health sciences, and nor do students receive a grounding in core subjects.

*“In my first year, I hadn’t done chemistry in eleven years [of schooling] … … I was struggling in this module and when I went to consult my chemistry lecturer, I was told if I didn’t understand the basic concepts of chemistry then I chose the wrong career …*” (Student).

“*… I am doing my second year I repeated my second year …. they gave me another chance although I had to explain why I failed …”* (Student).

### Rural-urban migration

The students pointed out that people, especial young people, migrate to cities as a result of the under-development of rural areas. This migration comes with the culture shock of new experiences and learning new habits in order to make their way in the new environment. Most students said that they found the change difficult.

*“… there are not even traffic lights in KwaNongoma and when you come here there are a thousand of them …. , you just wonder how to cross this road.”* (Student).

Going to a tertiary institution came with unexpected changes and challenges.

“*During all my basic education I used one classroom, or if we had to move, we were escorted by a teacher … … when I got to university, I was now expected to move from one lecture hall to another …*.” (Student).

The rural students had more difficulty than most students in adjusting to university life, having lived in relatively closed communities. They were accustomed to being with friends in familiar places, and now they had to find their way about the university alone.

*“In my first week at university I missed almost all the morning classes as I was expecting to hear the bell ring, but this was not the case here* …” (Student).

### The UYDF intervention

The students expressed gratitude to the UYDF for understanding their need for more than what a scholarship usually provides.

*“… given such an opportunity all I can do is study and pass and become the best radiographer …. This is not just bursary, the programme offers more and we are like a family*.” (Student).

The UYDF management realised that it is not worth spending a lot of money on students without giving proper support to assist them overcome the challenges they face at tertiary institutions.

### Home away from home

The students develop a close relationship with the UYDF. Participants stated that they feel cared for, as all their needs were taken care of by the UYDF. The students that were assisted by the UYDF included: students who were orphans and others who came from broken families who had feelings of loneliness and hopelessness, and they benefited most from this support and encouragement. UYDF appointed a chief mentor to keep track of the students, and the students had to account to him when there were difficulties.

*“… Umthombo [UYDF] is like home to me. Any time you can call if you have a problem with money, the mentor is there to take you through, and they always help you ….”* (Student).

Most of the participants have not previously been away from home and this requires adjustment, as they now have to adjust to seeing their families only during holidays. The majority are affected by homesickness especially in their first few months at IHLs, but with time they do adjust, although not without personal stress [21, 30]. The students’ social and academic adjustment has implications for the overall success of the student. Failure to do this often delays graduation and some students drop out, thus UYDF is playing a pivotal role in making sure that students adjust to both social and academic life.

### Post study placement anxiety

The students remarked on the fact that UYDF goes beyond providing a scholarship as it further facilitates placement of their graduates.

*“…. I would say the UYDF made a good plan by signing the MOU [memorandum of understanding] with Department of Health, because as UYDF graduates we have no problem of finding jobs, unlike many graduates in South Africa”* (Student).

*“…. People who graduate are not guaranteed jobs in South Africa. UYDF graduates are kind of lucky because of the MOU with the Department of Health that tries to place the UYDF graduates back in their rural hospitals without any interviews ….”* (Student).

In summary, most students indicated that UYDF had broken the cycle of poverty by introducing career guidance, informing them of work opportunities in the health care field, and making it possible for them to complete the necessary courses. The scholarship scheme did more than just provide financial support but also helped to develop the individual’s coping skills through the mentorship programme that prepared them for life at university and away from home, and to cope during difficult times.

### Graduates

**Fig. 2:**
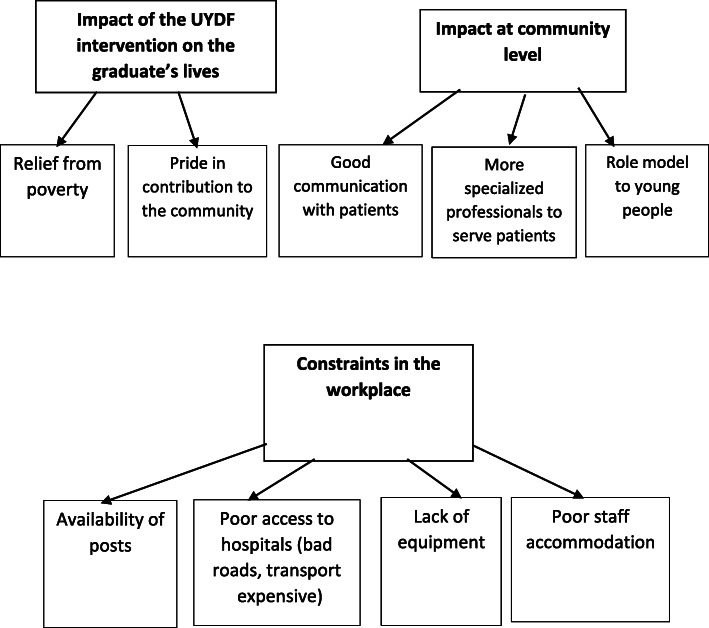
Graduates views regarding the impact of the UYDF intervention and the constraints on its effectiveness

The three FGD groups gave similar responses to the main topics introduced by the facilitator, namely (1) the impact of the UYDF intervention on the personal lives of the graduates, (2) the impact at community level; and (3) the difficult workplace environment. (see Fig. 2 below).

The graduates talked extensively about the way in which the scholarships changed their lives. They had been removed from a poverty-stricken situation.

*‘ … I come from a poor family and I never thought my family could afford tertiary education for me …. Without UYDF, I would not have made it this far …*. ’ (Graduate).

They said that the UYDF programme provided them with funds for studying as well as basic requirements for students. This freedom from pressing financial need continued after graduation because they now earn salaries. Most are now breadwinners for their families and can afford the basic needs of life.

*‘ … as a student I would budget my money and sometimes buy a few things for my siblings but now that I am working life has changed and whatever my family needs, I make sure I provide it. That’s how good UYDF has been for me...*’ (Graduate).

*“…. I love my community that much; I want to see it prosper and the only person to bring that change is me …*’ (Graduate).

Most of the graduates spoke of the pride they take pride in their contributions to others.

*‘ … We are willing to start a fund where we can put as little as R100 in future it will help other students who are in the same situation as we were …. Even if it goes towards their studies in other professions … … ’* (Graduate).

The graduates realised that although they are making a difference in the health sector, other professions such as teachers, engineers, architects are still needed in the rural communities in order to stimulate development in rural areas.

### The impact of UYDF at community level

Most of the participants mentioned the impact of the scholarship scheme at community level since it was introduced in 1999. Patients can now access health services without needing interpreters to convey the message to the health care practitioners.

*‘ … there are some things that my rural home people cannot explain clearly to a white doctor or nurse because they do not know how to say it in their language … .it is now easy to talk to us because they are able to say it in isiZulu’* (Graduate).

The increase in qualified health care workers in rural hospitals assists the patients who used to have to go to a regional or tertiary hospital for a small procedure. Previously some community patients could not travel to a distant facility because of the cost.

*‘ … it is no longer necessary for people in the rural areas to have appointments scheduled in the peri-urban / urban parts of the region because now you also find an optometrist and audiologist at a rural hospital which was usually not the case before … ’*, (Graduate).

Some participants noted that they had become role models for rural youth. They said that young people do not value education and drop out of school because they do not see the value of continuing. These local graduates can act as a point of reference for other youth facing similar challenges.

*‘ … Umthombo (UYDF) has afforded me an opportunity which my family was not able to because I come from a very poor background … , now children in my community will see the importance of education.’* (Graduate).

*‘ … I have built my mother a house and I now drive …. , most people in my community also wish to do the same for their families as well as for themselves’.* (Graduate).

Some graduates believed the current marketing strategy of that UYDF is insufficient and this needs to be strengthened to reach more learners at school as early as possible.

*‘ … Umthombo (UYDF) should go out there into schools and make the staff and students know the services they provide, what programmes they sponsor, all that information is not readily available … ’* (Graduate).

### Difficult workplace environment

Despite the gains in health care provision afforded by the scheme, several difficulties remained which made it difficult for the rural hospitals to retain staff. The graduates said that even though they were from area, the reality of the poor infrastructure in rural communities may affect what the UYDF’s programme is trying to change. There were several examples of poor infrastructure.

*‘ … as much as we want to stay long in our rural hospitals but we sometimes feel that we are not appreciated if you look at the type of accommodation that is available within the hospital’ … … it is difficult to work in a place where you are not appreciated’.* (Graduate).

The availability of a sufficient number of posts in rural hospital and good equipment is essential for the smooth running of the system and the job-satisfaction and well-being of the staff. If there are insufficient posts or if the existing posts are not filled, over-worked staff may eventually leave. These limitations also affect the variety of services that are required. Generally, there is a lack of equipment in public hospitals which affects the delivery of quality health services to the community.

*‘ …. the rural community also deserves better and quality health care, as this is stipulated by the constitution of South Africa’ …. sometimes there are essential posts that are not available at district hospital ….’* (Graduate).

*‘In my first year at work I had to work the whole year without proper equipment … this affected the provision of quality services as I had to compromise a lot’* (Graduate).

Graduates pointed out that long distances between the location of the health care facilities still exists in most rural communities. For health service providers the poor transport and inadequate roads affects their mobility between clinics and hospitals and risks the lives of their patients.

*‘The accessibility of health care facilities in rural areas still remains (a problem), there is not much of choice in relation to health care facilities and if they are there, they are isolated …. ’* (Graduate).

*‘Transport – the availability of transport is a major challenge in our area, the roads are terrible and sometimes not usable …. ’* (Graduate).

Because some of the graduates have decided to get married or engaged to local people, this works in favour of stabilising the hospital workforce and is an unintended consequence of the UYDF policy of requiring graduates to return to their area of origin. Others are marrying fellow UYDF graduates and this results in them staying longer than the contract period. The graduates thought that if the UYDF had a bigger workforce the graduates would have an opportunity to involve the UYDF on issues affecting the hospitals where they are placed. Due to the difficulties faced in these workplaces some graduates would like to work in the private sector, but because they are indebted to UYDF they feel obliged to stay.

### Management

**Fig. 3:**
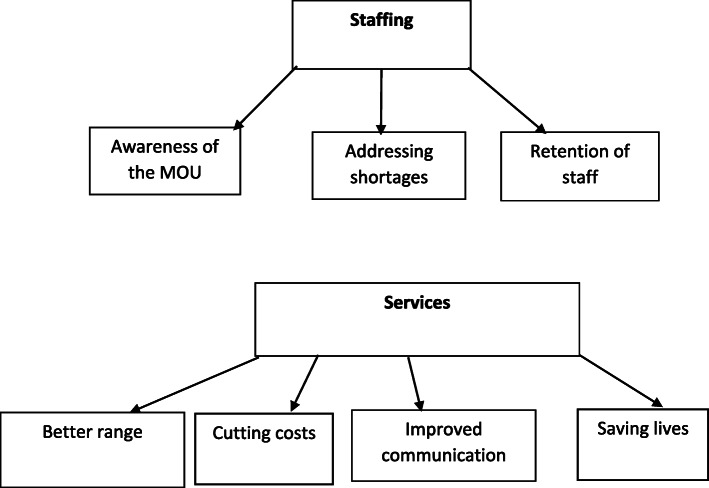
Managers views on the effectiveness of the UYDF intervention in addressing staff shortages and poor service delivery in rural areas

The health managers dealt with two main topics, their problems with finding and retaining staff, and the changes which have taken place in their hospitals as a result of the UYDF programme. (see Fig. 3 below).

### Staffing

#### Awareness of UYDF services

The UYDF had signed a memorandum of understanding (MoU) with the provincial Departments of Health but some of the hospital managers were not aware of this agreement and what it means to them and their facilities.

*“We were told by UYDF staff that there is a MoU between the department and them (UYDF), but no one has ever explained these agreements to us as the hospitals, and we always get graduates allocated to our hospital by the province ….” .* (HR Manager).

*“If the province can give details on the graduates they are allocating to us, if we knew they were from UYDF, that will help us allocate their duties, and let the UYDF Graduates do more of the outreach programmes because of their local knowledge of the area and community”.* (Medical Manager).

However, most managers knew about the UYDF graduates and that they had played a part in different areas of hospital functioning, and this had been welcomed by both the hospital community and the community at large. The managers reported that the UYDF graduates were enthusiastic in their support of community development, which helped with the prevention of diseases.

#### Addressing shortage changes due to the UYDF programme

The respondents said that not only was there a general scarcity of health care professionals in the country, but few qualified personnel chose to work in rural areas. Most said that the difficulty of attracting health care professionals to rural hospitals was ongoing and the rural hospitals where this study was conducted were severely affected. There had been vacant posts in some hospitals for 20 years and district hospitals reported being unsuccessful in recruiting and retaining a staff for the posts.

“*When I started here there was only one doctor and one clinical associate with 6 wards to be covered (Male, Female, Paediatrics, Maternity, OPD and Casualty). Patients used to go back home untreated, and sometimes the ward rounds would only be done once a week in order to cover all wards and that increased the death rate at our hospital.”* (Medical Manager).

“… *Our facilities have struggled in the past to train and attract professionals especially health care professionals, due to the lack of funding and the geographical location …”* (District HR Manager).

The UYDF has produced over 300 graduates covering seventeen different health science disciplines over a period of 19 years and this has helped to staff sixteen different hospitals. The Managers reported that the UYDF graduates had brought some stability in terms of reliable staff, although there are still not enough of them. Some said that the posts in their institutions were filled only when the UYDF scholarships started to produce graduates who returned to work back their commitments in their local hospitals. This was not always the case because some staff get married and want a better education for their children, so they leave the rural hospitals prematurely. Also, the workload may become too heavy and these factors leaving more likely.

“… *Majority of the healthcare professionals at our institution are there through UYDF. They are long-term staff that bring stability”.* (CEO).

### Retention:

“… *UYDF assists with our staff retention strategy as the students work in their rural communities after completion of their studies, and the majority of them stay longer than their contracts….*”. (CEO)

However, some graduates do not choose to work in rural areas because of the lack of equipment and the poor accommodation for doctors.

*“…. Even those who came to our facilities, we are unable to retain them, they are overworked with no recreational facilities around our hospitals …”* (HR Manager).

### Services

The medical manager in one of the hospitals said that the services have improved in their hospitals, because of the medical practitioners from UYDF. Even though the staffing is not at an optimal level it is better than it was, and they will continue to receive staff through the UYDF. The hospitals that had been working with UYDF for a longer period had received a larger number of healthcare stuff than those with fewer years.

The respondents mentioned a variety of advantages to employing local health care practitioners:

### Better range of services and cutting costs

*“… it is no longer necessary for people in the rural areas to have appointments scheduled in the regional or tertiary hospitals …. because patients get a variety of services at the local hospital, such as psychological services, eye care and all rehabilitation services are on site now, which was not the case before …”* . (Medical Manager).

This variety of services has helped rural hospitals to cut the cost of transporting the patients to referral hospitals.

#### Improved communication, saving lives

The medical officers pointed out that patients can communicate in isiZulu with the UYDF graduates, who, in turn, understand the community needs and are willing to go the extra mile to provide quality health care. This not only helps to produce a stable workforce but makes it possible for the hospitals to have outreach services to prevent the spread of diseases in the community.

*“They are generally local and know the language and situation of the patients”.* (CEO).

*“Majority of the UYDF graduates had a strong sense of commitment to the rural communities where they work, and they showed responsibility in giving back to their communities of origin ….”* (Medical Manager).

*“No one wants to see anyone die, and an idea that fights mortality is always a good idea which needs to be supported. This is exactly what the UYDF is doing for our institution – it prevents deaths of many people”.* (Medical Manager).

## Discussion

This paper explores the improvements in health service delivery as perceived by the beneficiaries of the bursary and mentorship scheme in a rural area of the province of KwaZulu-Natal in South Africa, and the health service managers from the area, and the impact of the scheme on the lives of the participants and the communities in which they live. Ultimately the goal is to argue for the replication of the intervention in other rural areas both within South Africa and beyond its borders.

There was considerable overlap in the accounts given by graduates and health managers in explaining the ways in which the health system in the area had changed qualitatively: the health professionals were not so overworked because there were more of them, filling posts which were unattractive to applicants from outside the area. They offered a wider range of services at one hospital, reducing the need for transporting patients to other centres for treatment, a process which was expensive and resulted in delays in treatment. The rural hospitals now have optometrists, radiographers, dieticians, and audiologists – professions which were previously only found only in urban areas [30–32]. The UYDF graduates have the advantage of knowing the area well and understanding the language, which makes them better able to communicate with patients [16, 33], and to take on outreach work. The health professionals believed that more lives were being saved in hospitals where the UYDF scheme operated. Importantly, graduates are more likely to be retained in the service, thus stabilising the hospital services. There were constraints to their effectiveness as public servants: lack of essential equipment, poor accommodation for doctors on call, long distances between hospitals and outreach clinics and poor roads connecting them.

The results distilled from the interviews and focus group discussions [34] with the students and graduates demonstrated that the UYDF scheme does more than that: it can contribute to the general development of the communities where it operates. Young people who would otherwise have added to the larger numbers of unemployed people in the area are now gainfully employed, supporting their families and acting as role models to learners at school, encouraging them to choose school subjects wisely, to work harder and be more ambitious in their choice of career. Because they were likely to remain in the area, they were more committed to community development.

The UYDF bursary scheme worked well because the components were integrated, from the early career guidance talks in the school, through financing of all aspects of their study years and mentoring both socially and academically which was thought to reduce the number of years needed to complete their degrees. The collaboration with the Department of Health was a key component from the selection of students by hospital committees so that hospital managers chose students that would suit their needs and made employment opportunities for them during the University vacations. They liaised with the provincial authorities so that posts were available when the students graduated, and a majority of the graduates stayed permanently in the area.

The study identified problems arising from the general poverty and underdevelopment of the area. The schools were of poor quality, the students were ill-prepared academically and given little guidance on tertiary education choices. An effective education system can contribute to rising income and higher productivity [35, 36] and, in the case of South Africa, a review of the effects of education on a range of development outcomes for the period 1960–2010, highlighted the positive effects of tertiary education on income growth [37, 38].

The other caveat raised in all the focus groups was the need for reform in the health system if the UYDF intervention is to be optimally effective. Some South African government strategies such as the proposed National Health Insurance (NHI) and the Human Resources for Health Strategy [39], acknowledge deficiencies in the system. The hospitals were not well equipped for the young professionals so that audiologists, for example, were not immediately effective when the equipment they needed was not available; buildings were not well maintained; the road network is the area was not well-developed or maintained. All of these shortcomings limited the effectiveness of the professional staff and contributed to the difficulty that rural hospitals have in retaining staff.

Ultimately the accounts from the people involved in the UYDF intervention have shown how a similar intervention replicated in other rural areas in countries in the SADC region, would, in time, produce better health services throughout southern Africa. If these were coupled with better management of basic education systems and other government infrastructure, economic development of rural areas would benefit. Many areas in Africa need effective development initiatives and this innovative programme demonstrates the feasibility of initiating such a programme, the benefits and the potential for sustainability.

## Conclusions

The UYDF has designed a system that was well thought-out and is achieving its goal of improving health services in underdeveloped rural areas of South Africa. More could be achieved if other government services in the areas were simultaneously improved and if the system were replicated elsewhere. The students and graduates from rural areas are involved in sustaining health services in rural areas, while rural managers support the programme and made suggestions for improvements and the need to promote the programme in other regions.

## Supplementary Information

**Additional file 1.**

## Data Availability

Data and materials supporting the results are available at the Umthombo Youth Development Foundation (UYDF) and accessible upon request at https://www.umthomboyouth.org.za/and the Director of the UYDF can be contacted at gavin@umthomboyouth.org.za for any information that is not available on the website.

## Abbreviations

FGD: Focus Group Discussions
HCP: Healthcare Professionals
MBChB: Bachelor of Medicine and Bachelor of Surgery
MoU: Memorandum of Understanding
NHI: National Health Insurance
NPO: Non-profit organisation
OPD: Out-Patient Department
SADC: Southern African Development Community
SSRA: Social Science Research Assistant
UYDF: Umthombo Youth Development Foundation
