# Supplementary material for: Engaging future healthcare professionals for rural health services in South Africa: students, graduates and managers perceptions
Source: BMC Health Serv Res. 2021 Mar 12;21:220. doi: 10.1186/s12913-021-06178-w (PMC7953823; doi:10.1186/s12913-021-06178-w)
Supplement: Supplementary file 1 — Additional file 1. [file 12913_2021_6178_MOESM1_ESM.docx]

**Appendix 1:** **Interview guide – Students**

**Research Topic:**

**SKILLS DEVELOPMENT: ROLE OF UMTHOMBO YOUTH DEVELOPMENT FOUNDATION (UYDF) IN THE HEALTHCARE SKILL SHORTAGE IN RURAL SOUTH AFRICA**

**Demographic details**

Name & Surname: _________________________________________________

Age: ___________________ Gender: __________________________

Place of origin: ___________________________________________________

Marital status: ____________________________________________________

Number of dependents: __________

Other income source: _____________________________________________

Parents’ occupation: ______________________________________________

Place of interview: ________________________________________________

Name of University: _______________________________________________

Name of degree: _________________________________________________

Career aspiration: ________________________________________________

**Questions**

1. In a few sentences, please describe what you know about UYDF and how you came in contact with them?
2. As a current student, how are you benefiting from UYDF?
3. Is there any additional thing that UYDF should be doing to assist you during your studies?
4. How has the UYDF affected your personal life?
5. What other contributions has the UYDF has made to your life?
6. How can you describe your life/ and that of your family before you became beneficiary of the UYDF support?
7. What other life plans would you have adopted if UYDF did not offer you this financial support?
8. What are your plans after this degree?
9. What were the conditions stipulated in your agreement with the UYDF for receipt of the bursary?
10. Would you consider breaching this agreement if you were offered a high paying job after your degree? If yes / no, please explain why?
11. How do you plan to give back to UYDF after your graduation?
12. Do you know any other contribution that UYDF has made to your community / healthcare facility that you can share with us?
13. Is there any recommendation that you would make to the government or the National Department of Health in support of the work of UYDF?
14. Any other thing that you would like to say about UYDF that we have not mentioned above?
15. Would you advise your friends or family members to apply for UYDF funding to become a health professional? If yes, please explain:

Thank you for your participation in this study.

**Appendix 2:** **Interview guide (Qualitative data) – Graduates**

**Research Topic:**

**SKILLS DEVELOPMENT: ROLE OF UMTHOMBO YOUTH DEVELOPMENT FOUNDATION (UYDF) IN THE HEALTHCARE SKILL SHORTAGE IN RURAL SOUTH AFRICA**

**FOCUS GROUP DISCUSSIONS: A GUIDE**

| FGD facilitator’s name |  |
| --- | --- |
| Date of FGD |  |
| Venue |  |
| Total number of participants |  |
| Start time and end time of the FGD: |  |

**Baseline information**

1. Did you all use UYDF bursary to complete your studies?
2. What were the good and bad experiences with using UYDF bursaries at your tertiary level?
3. How long has it been since each participant graduated?
4. How long have you been serving at your current health facility?

**UYDF Impacts**

1. How can you describe your lives before the studies and now?
2. What are the changes that you have observed in your lives since you started working?
3. What are other direct or indirect effects of UYDF that you are aware of?
4. What are the impacts that you have made to your host institutions/ facilities?
5. Would you say that UYDF is a good model to improve rural HCPs shortage and service delivery? If yes, please say why?
6. How would you give back to UYDF if you were able to do so?
7. Are there some recommendations to the government, UYDF or the DOH regarding the UYDF model?
8. Please list any other contribution that UYDF has made in your life, community or DOH that we did not mention above?

Thanks for your participation in this research study.

**Appendix 3:** **Interview guide (Qualitative data) – Hospital & Districts Leaders**

**Research Topic:**

**SKILLS DEVELOPMENT: ROLE OF UMTHOMBO YOUTH DEVELOPMENT FOUNDATION (UYDF) IN THE HEALTHCARE SKILL SHORTAGE IN RURAL SOUTH AFRICA**

**Demographic characteristics** **of participants**

Name & Surname: ……………………………………………………………………………………….

Gender: …………………………………… Age: ………………………………………………….

Name of district: …………………………………………………………………………………………

Position / Title in the Healthcare Facility / District office: ...……………………………………………

Period in this position (in years): ….……………………………………………………………………

Number of staff reporting to you: ……………………………………………………………………..

**Related questions**

1. What has been the situation with healthcare professionals (HCP) stuff shortage in your hospital or district facilities?

|  |
| --- |

2. Are you aware of UYDF and its role in supporting rural-based healthcare facilities with stuffing? If yes, please provide a description

|  |
| --- |

3. How many UYDF graduates have you received since the start of their placement and what are their impacts on your facilities’ health services delivery?

|  |
| --- |

4. Could you describe the situation before and after the UYFD graduate joined your district facilities? i.e What has changed since their arrival?

|  |
| --- |

5. What other impacts have they made to the rural-based healthcare facilities at large?

|  |
| --- |

6. Any other comment about the role and impact of UYFD graduates in the DOH?

|  |
| --- |

I thank you for the time you have given and participation in this research study!
